# TNF-α Carried by Plasma Extracellular Vesicles Predicts Knee Osteoarthritis Progression

**DOI:** 10.3389/fimmu.2021.758386

**Published:** 2021-10-06

**Authors:** Xin Zhang, Ming-Feng Hsueh, Janet L. Huebner, Virginia B. Kraus

**Affiliations:** ^1^ Duke Molecular Physiology Institute, Duke University School of Medicine, Duke University, Durham, NC, United States; ^2^ Department of Orthopaedic Surgery, Duke University School of Medicine, Duke University, Durham, NC, United States; ^3^ Department of Medicine, Duke University School of Medicine, Duke University, Durham, NC, United States

**Keywords:** extracellular vesicles, knee osteoarthritis progression, immune cells, cytokines, TNF-α

## Abstract

**Objectives:**

To identify plasma extracellular vesicles (EVs) associated with radiographic knee osteoarthritis (OA) progression.

**Methods:**

EVs of small (SEV), medium (MEV) and large (LEV) sizes from plasma of OA participants (n=30) and healthy controls (HCs, n=22) were profiled for surface markers and cytokine cargo by high-resolution flow cytometry. The concentrations of cytokines within (endo-) and outside (exo-) EVs were quantified by multiplex ELISA. EV associations with knee radiographic OA (rOA) progression were assessed by multivariable linear regression (adjusted for baseline clinical variables of age, gender, BMI and OA severity) and receiver operating characteristic (ROC) curve analysis.

**Results:**

Based on integrated mean fluorescence intensity (iMFI), baseline plasma MEVs carrying CD56 (corresponding to natural killer cells) predicted rOA progression with highest area under the ROC curve (AUC) 0.714 among surface markers. Baseline iMFI of TNF-α in LEVs, MEVs and SEVs, and the total endo-EV TNF-α concentration, predicted rOA progression with AUCs 0.688, 0.821, 0.821, 0.665, respectively. In contrast, baseline plasma exo-EV TNF-α (the concentration in the same unit of plasma after EV depletion) did not predict rOA progression (AUC 0.478). Baseline endo-EV IFN-γ and exo-EV IL-6 concentrations were also associated with rOA progression, but had low discriminant capacity (AUCs 0.558 and 0.518, respectively).

**Conclusions:**

Plasma EVs carry pro-inflammatory cargo that predict risk of knee rOA progression. These findings suggest that EV-associated TNF-α may be pathogenic in OA. The sequestration of pathogenic TNF-α within EVs may provide an explanation for the lack of success of systemic TNF-α inhibitors in OA trials to date.

## Introduction

Extracellular vesicles (EVs) are released by almost all mammalian cells. Due to their cargo (cytoplasmic proteins, DNA, mRNA, miRNA, small non-coding RNAs, mitochondria, and cytokines), EVs are believed to be able to act as mediators of cell-to-cell communication and as paracrine effectors (1–4). Studies in OA have focused on the beneficial effects of mesenchymal stem cell (MSC)-derived EVs (5, 6), the detrimental effects of subchondral bone osteoblast-derived small EVs (SEVs) (7), and the surface markers and cytokine cargo of SEVs (also known as exosomes) in OA synovial fluid (SF) (8, 9). SEVs from knee OA SF carry surface markers CD9, CD81 and CD63 and cytokines (IL-1β, IL-2, IL-4, IL-5, IL-6, IL-13, IL-17, TNF-α and IFN-γ (8–10), and are associated with OA disease severity (8). SEVs from OA SF induce the release of pro-inflammatory cytokines (IL-1β, IL-6 and TNF-α), chemokines and metalloproteases *in vitro* by M1 macrophages (9), enhance chemotaxis of peripheral blood mononuclear cells, promote inflammatory responses, and inhibit chondrocyte proliferation (10). SEVs from IL-1β stimulated human synovial fibroblasts significantly up-regulate articular chondrocyte expression of MMP-13 and ADAMTS-5 (11). Taken together, these studies suggest a pathogenic role of SF-derived EVs in OA.

Despite the evidence for a role of EVs in the pathogenesis of OA, to our knowledge, there have been no previous studies evaluating the role of EV subpopulations in OA progression. To fill this important knowledge gap, we profiled OA and healthy control (HC) plasma for EV surface markers, ‘endo-EV’ (within EV) cytokine cargo, and exo-EV cytokine concentrations (in EV-depleted supernatant after ExoQuick precipitation of EVs from the unit of plasma), to evaluate and compare their associations with knee radiographic (r)OA severity and progression.

## Methods

### Study Participants

Sixty plasma specimens were analyzed including: (1) healthy controls (HCs, n=16), non-progressive OA (OA-NP, n=16) and progressive OA (OA-P, n=14) from the completed Genetics of Generalized Osteoarthritis (GOGO) study (12); and (2) additional HC samples (n=6) from the commercial vendor (Zenbio). The samples were matched for gender, race and decade of age (**Table 1**). Samples were stored at -80°C until analysis. All samples and data were acquired with informed consent under IRB approval of Duke University or the commercial vendor (Zenbio).

**Table 1:** Demographic information of the study participants.

	HC*	HC	OA-NP	OA-P
Sample number	n=6	n=16	n=16	n=14
Mean (SD) age at enrollment (years)	55.5 (11)	68.4 (8)	68.7 (8)	69.3 (8)
Gender (Female) %	50%	50%	50%	57%
Mean (SD) BMI at enrollment (kg/m^2^)	28 (8)	27 (3)	32 (8)	29 (5)
Median (range) Summed Baseline K/L grade	N/A	0 (0)	2 (1-6)	2 (1-6)
Median (range) Summed Change in K/L grade	N/A	0 (0)	0 (0)	3 (1-6)

HC, healthy control; *obtained from Zenbio; OA-P, radiographic knee osteoarthritis progressor; OA-NP, radiographic knee osteoarthritis non-progressor; SD: standard deviation; BMI, body mass index; K/L grade, Kellgren and Lawrence grade; N/A, not applicable.

### Radiographic Procedures and Grading

Knee radiographic imaging was performed as reported previously for the GOGO study (12) and scored for Kellgren and Lawrence (K/L) grade (0–4) (12–14). K/L scores from both knees of a participant were summed yielding scores with range 0-8. HCs from GOGO were defined as participants having knee K/L grade 0 bilaterally. Participants having knee OA was defined as having summed K/L grade ≥1 at baseline. The change of K/L scores from both knees of a participant were summed yielding scores with range 0-6. Radiographic knee OA progression was defined as K/L grade increase ≥1 unit in at least one knee during follow-up (mean 3.8 years, range 1.1-8.6 years) (**Table 1**). HCs from Zenbio were defined as no self-reported diseases or medical conditions.

### EV Separation From Plasma Samples

EV isolation required 50 μl plasma for each marker panel as previously reported (4, 15). Blood samples were centrifuged at 3000 rpm for 15 min at 4 °C to separate plasma from cells and debris; plasma samples were aliquoted and frozen at − 80 °C until analysis. Frozen plasma samples were thawed followed by centrifugation at 2000 g for 10 minutes at 4°C to remove remaining debris. EVs in plasma were separated by ExoQuick (System Biosciences) following the manufacturer’s instructions (4, 15, 16). As described below, EVs were profiled for surface markers and cytokines; endo-EV and exo-EV cytokine concentrations were also measured.

### Profiling EV-Carried Surface Markers and Cytokines by High Resolution Multicolor Flow Cytometry

As previously described (4, 15), EVs were profiled for the following surface markers to identify EV subpopulations by cell of origin (**Supplementary Table 1**) **(** 4, 15, 17–25): CD81, CD9, CD29, CD63, CD8, CD68, CD14, CD56, CD15, CD235a, CD41a, CD34, CD31, major histocompatibility complex (MHC)-class I antigens HLA-A, HLA-B and HLA-C (HLA-ABC), MHC-class II antigens HLA-DR, -DP and -DQ (HLA-DRDPDQ) (BD Biosciences), CD4, CD19 and MHC-class I antigen HLA-G (ThermoFisher Scientific). We also profiled endo-EV cytokines IL-1β, TNF-α, IFN-γ (BD Biosciences), and IL-6 (ThermoFisher Scientific). The percentages (%) and geometric mean fluorescence intensity (MFI) of EVs carrying each tested marker were determined using a high-resolution multicolor BD LSR Fortessa X-20 Flow Cytometer with the BD FACSDiVa software (BD Biosciences). The integrated MFI (iMFI) of surface markers and cytokines was calculated by multiplying percentage of positive population with the MFI of that population (15, 26, 27).

### Multiplex ELISA for Cytokine Quantification

To confirm the efficiency of the EV precipitation, the concentration and size distribution of particles (in the ExoQuick precipitate of EVs and the remaining EV-depleted supernatants) derived from 50 µl plasma were measured by nanoparticle tracking analysis and dynamic light scattering as previously reported (4, 15) (**Supplementary Figure 1**). As previously described (15), EV pellets were lysed in NP40 lysis buffer (Thermo Fisher Scientific) in the same volume as the EV-depleted supernatants. The concentrations of endo-EV and exo-EV (remaining EV-depleted supernatant after ExoQuick precipitation) cytokines were measured by multiplex ELISA using the Custom Pro-inflammatory Panel (IL-1β, IL-6, TNF-α, IFN-γ, Meso Scale Diagnostics) following the manufacturer’s instructions (15, 28, 29).

### Receiver Operating Characteristic (ROC) Curve Analysis

Multivariable logistic regression and ROC curve analyses (30, 31) were performed to evaluate the discriminant ability of baseline EV-related variables for knee OA progression, defined as change in knee K/L grade (summed across knees). Model stabilities were validated using a 2,500 non-parametric bootstrap resampling approach; 95% bias-corrected confidence intervals (CIs) for area under the ROC curve (AUC) are reported. Likelihood ratio (32) test was used to assess model fit; the R-square value (RSq) (33), corrected Akaike’s Information Criterion (AICs) (34) and the Bayesian Information Criterion (BIC) (35) are reported. Rsq closer to 1 indicates a better fit to the data; while for AIC and BIC, the model having the smaller value is considered better. Specificity was determined at sensitivity 80%. The analyses were performed using JMP Pro 15 (SAS). AUC is interpreted as follows: AUC ≤0.5 indicates no better than a random classifier; AUC >0.5 is considered validated; AUC >0.65 is considered a moderate discriminant capability.

### Statistical Analyses

Data in this study were not normally distributed based on D’Agostino-Pearson omnibus normality test; therefore, nonparametric analyses were performed. Comparisons between HC, OA-NP and OA-P were performed using Kruskal-Wallis test. Comparisons of endo-EV and exo-EV cytokines were performed using Wilcoxon matched-pairs signed rank test. Comparisons between the tested cytokines in each participant group were performed using Friedman test. False Discovery Rate (FDR) was generated using the Benjamini and Yekutieli method with significant results defined by FDR (q value) <0.05. Multivariable linear regression modeling was performed with adjustment for baseline clinical variables (age, gender, body mass index [BMI] and summed knee OA K/L score) to identify associations of endo-EV and exo-EV biomarkers with knee rOA progression, defined by change in summed K/L score from baseline to follow-up. A p value <0.05 was considered statistically significant. GraphPad Prism 8.0 software (GraphPad) and JMP Pro 15 software were used for statistical analyses.

## Results

### Multiple Immune Cell-Related LEVs and MEVs Were Associated With Knee rOA Progression

There are currently no specific markers for different EV subtypes (36). Following the recommendations from the International Society for Extracellular Vesicles (36), we used operational terms (range of sizes along with descriptions of cell of origin defined by their surface markers) to describe EV subsets as in our previous work (4, 15). With our newly developed high-resolution multicolor flow cytometry-based methodology, we identified three major subsets of plasma EVs in human HCs: large EVs (LEVs), 1000-6000 nm; medium sized EVs (MEVs), 100-1000 nm; and SEVs, <100 nm; these major subsets based on size were confirmed using dynamic light scattering (4, 15). In this study, these three major subsets of plasma EVs were also identified in plasma of participants with knee OA. Plasma EVs from HC and OA participants all carried surface markers of human stem cells and progenitor cells, immune cells, activated pro-inflammatory fibroblasts, epithelial and endothelial cells indicative of their cell origins (**Supplementary Figure 2** and **Supplementary Table 1**) **(** 4, 15, 17–25).

**Figure 1:**
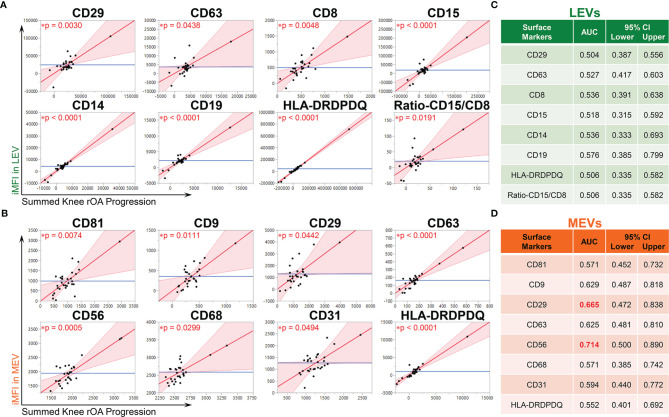
Multiple immune cell-associated LEVs and MEVs at baseline were associated with knee radiographic (r)OA progression. Plasma EVs from OA participants (n = 30, 53% OA-NP and 47% OA-P) at baseline were profiled with the indicated surface markers by high resolution multicolor flow cytometry. **(A, B)** Multivariable linear regression modeling was performed with adjustment for the baseline clinical variables (age, gender, BMI and summed knee OA K/L score) to identify the associations of the knee OA radiographic severity changes from baseline to follow-up with the baseline iMFI of each surface marker in gated LEVs, MEVs or SEVs. The Leverage Plots represent plots of leverage of summed knee rOA progression (x axis, scaled in units of the response) vs the baseline iMFI (y axis) of the indicated surface markers in LEVs **(A)** and MEVs **(B)** with an asterisks indicate the p < 0.05. No SEV subpopulations based on EV surface markers were associated with knee rOA progression. **(C, D)** Multivariable logistic regression and ROC curve analysis were used to assess AUC of the knee rOA progression-associated surface markers in LEVs **(C)** and MEVs **(D)** to predict progression of radiographic knee OA. Since all tested surface markers in SEVs were not significantly associated with knee rOA progression, they were excluded from ROC analysis. AUC > 0.5 was considered validated and AUC > 0.65 (red font) was considered a moderate discriminant capability. 95% bias-corrected confidence intervals (CIs) for AUCs are reported. LEVs, large EVs; MEVs, medium EVs; iMFI, integrated mean fluorescence intensity. *p < 0.05.

Baseline iMFI of multiple plasma EV subpopulations were associated with baseline clinical variables including age, gender, BMI and summed knee OA K/L score (**Supplementary Figure 3**). Adjusting for these baseline clinical variables, baseline iMFI of multiple plasma LEVs and MEVs were statistically significantly associated with knee rOA progression. LEV subpopulations associated with rOA progression included the following: CD29^+^, CD63^+^, CD8^+^, CD15^+^, CD14^+^, CD19^+^, and ratio of CD15/CD8 (reflecting neutrophil-EV to T cell-EV ratio) (**Figure 1A**). MEV subpopulations associated with rOA progression included the following: CD81^+^, CD9^+^, CD31^+^, CD29^+^, CD63^+^, CD56^+^, CD68^+^, and HLA-DRDPDQ^+^ (**Figure 1B**). No SEV subpopulations based on EV surface markers were associated with knee rOA progression. These LEV and MEV surface markers yielded AUCs >0.5 (**Figures 1C, D** **)**; CD56^+^ MEVs (AUC 0.714), corresponding to NK cell-EVs, yielded the highest AUC (**Figure 1D**).

### Endo-EV TNF-EV TNF-α in Plasma Associated With Knee OA Progression

**Figure 2:**
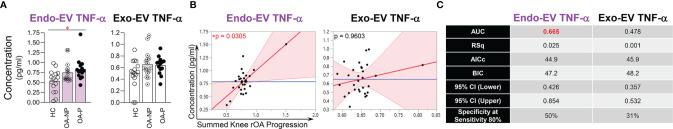
The concentration of endo-EV TNF-α at baseline was associated with knee rOA progression. The concentrations of exo-EV and endo-EV TNF-α in plasma from HC (n = 16), OA-NP (n = 16) and OA-P (n = 14) participants at baseline were measured by multiplex immunoassay. **(A)** The graphs represent the differential concentrations of endo-EV and exo-EV TNF-α between HC, OA-NP and OA-P. Comparisons between HC, OA-NP and OA-P were performed using Kruskal-Wallis test with significant results defined by FDR q< 0.05; asterisks indicate the p value as * <0.05. **(B)** Multivariable linear regression modeling was performed with adjustment for the baseline clinical variables to identify the associations of summed knee rOA progression with baseline concentrations of TNF-α in OA participants (n = 30, 53% OA-NP and 47% OA-P). The Leverage Plots represent plots of leverage of summed knee rOA progression (x axis) vs the baseline concentrations (y axis) of endo-EV and exo-EV TNF-α. **(C)** Multivariable logistic regression and ROC curve analysis were used to assess AUC and specificity (at sensitivity 80%) of endo-EV and exo-EV TNF-α in OA participants (n = 30, 53% OA-NP and 47% OA-P) to predict progression of radiographic knee OA. Rsq, AICs, and BICs were based on likelihood ratio test. 95% bias-corrected confidence intervals (CIs) for AUCs are reported. Endo-EV, within EV; Exo-EV, outside EV; HC, healthy control; OA-P, radiographic knee osteoarthritis progressor; OA-NP, radiographic knee osteoarthritis non-progressor.

TNF-α is a classical pro-inflammatory cytokine playing critical roles in OA pathogenesis (29, 37–41). Recently, we found that endo-EV concentrations of TNF-α (in lysates of EV pellets) were significantly higher than the exo-EV concentrations (in EV-depleted supernatants) in the same unit volume of plasma from OA participants, suggesting that EVs are a major source of systemic TNF-α(15). Here, we report, the concentration of endo-EV TNF-α was significantly higher in OA-P than HC participants, while exo-EV TNF-α concentrations did not differ among the study groups (**Figure 2A**). Adjusting for baseline clinical variables, the baseline concentration of endo-EV TNF-α was statistically significantly associated with knee rOA progression (**Figure 2B**). In addition, the baseline concentration of endo-EV TNF-α predicted knee rOA progression with moderate discriminant ability (AUC 0.665, **Figure 2C**). In contrast, exo-EV TNF-α was neither associated with nor predictive of knee rOA progression (**Figures 2B, C** **)**. In addition to EVs being a major source of systemic TNF-α, this suggests that plasma endo-EV TNF-α represents a promising systemic biomarker for predicting risk of knee rOA progression.

**Figure 3:**
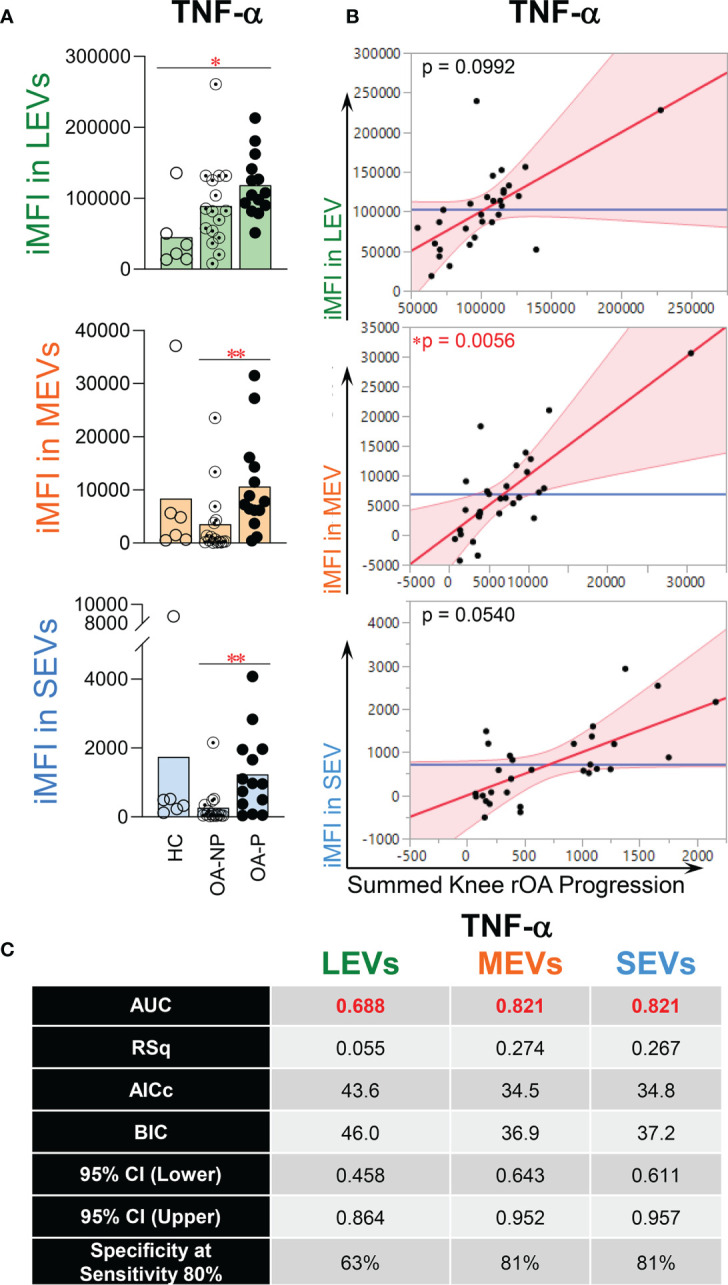
The iMFI of endo-EV TNF-α at baseline was associated with knee rOA progression. Plasma EVs from HC (n = 6), OA-NP (n = 16) and OA-P (n = 14) participants at baseline were profiled for intra-vesicle TNF-α by high-resolution multicolor flow cytometry. **(A)** The graphs represent the iMFI of TNF-α in gated LEVs, MEVs and SEVs between HC, OA-NP and OA-P. Comparisons between HC, OA-NP and OA-P were performed using Kruskal-Wallis test with significant results defined by FDR q< 0.05; asterisks indicate the p value as * <0.05 and ** <0.01. **(B)** Multivariable linear regression modeling was performed with adjustment for the baseline clinical variables to identify the associations of the knee OA radiographic severity changes from baseline to follow-up with the baseline iMFI of TNF-α in OA participants (n = 30, 53% OA-NP and 47% OA-P). The Leverage Plots represent plots of leverage of summed knee rOA progression (x axis) vs the baseline iMFI (y axis) of TNF-α in LEVs, MEVs and SEVs. **(C)** Multivariable logistic regression and ROC curve analysis were used to assess AUC and specificity (at sensitivity 80%) of TNF-α in LEVs, MEVs and SEVs in OA participants (n = 30, 53% OA-NP and 47% OA-P). Rsq, AICs, and BICs were based on likelihood ratio test. 95% bias-corrected CIs for AUCs are reported. LEVs, large EVs; MEVs, medium EVs; SEVs, small EVs; iMFI, integrated mean fluorescence intensity. HC, healthy control obtained from Zenbio; OA-P, radiographic knee osteoarthritis progressor; OA-NP, radiographic knee osteoarthritis non-progressor.

Plasma EVs from HCs, OA-NP and OA-P participants were assessed by flow cytometry for an array of cytokines including TNF-α. TNF-α was present in all sizes of plasma EVs and was the most abundant endo-EV cytokine of the four analyzed and the most abundant cytokine in all three participant groups based on mean percentage of TNF-α^+^ LEVs, MEVs and SEVs (**Supplementary Figure 4**). Baseline iMFI of TNF-α in LEVs was significantly higher in OA-P than HCs (**Figure 3A**), which is consistent with the differential concentration of endo-EV TNF-α observed between these two groups by ELISA based analyses (**Figure 2A**). Moreover, baseline iMFI of TNF-α in MEVs and SEVs was significantly higher in OA-P than OA-NP (**Figure 3A**). With adjustment for baseline clinical variables, baseline iMFI of TNF-α in EVs of all sizes was associated with knee rOA progression (P<0.1); this was statistically significant for TNF-α in MEVs (**Figure 3B**). Although the baseline iMFI of TNF-α in EVs of all sizes predicted knee rOA progression, MEVs and SEVs had higher AUCs and specificity (both AUC 0.821 and specificity 81% at sensitivity 80%) than LEVs (AUC 0.688 and specificity 63% at sensitivity 80%) (**Figure 3C**).

### Other Pro-Inflammatory Cytokines Associated With Knee rOA Progression

**Figure 4:**
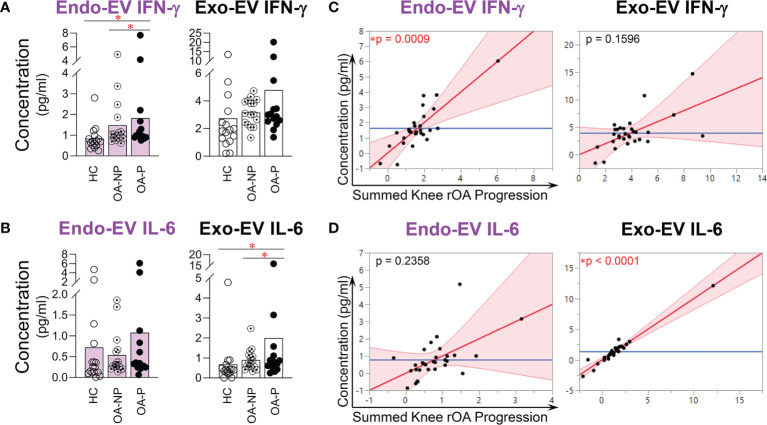
The concentrations of endo-EV IFN-γ and exo-EV IL-6 at baseline were associated with knee rOA progression. The concentrations of exo-EV and endo-EV IFN-γ and IL-6 in plasma from HC (n = 16), OA-NP (n = 16) and OA-P (n = 14) participants at baseline were measured by multiplex immunoassay. **(A, B)** The graphs represent the differential concentrations of endo-EV and exo-EV IFN-γ **(A)** and IL-6 **(B)** between HC, OA-NP and OA-P. Comparisons between HC, OA-NP and OA-P were performed using Kruskal-Wallis test with significant results defined by FDR q < 0.05; asterisks indicate the p value as * < 0.05. **(C, D)** Multivariable linear regression modeling was performed with adjustment for the baseline clinical variables to identify the associations of summed knee rOA progression from baseline to follow-up with the baseline concentrations of IFN-γ and IL-6 in OA participants (n = 30, 53% OA-NP and 47% OA-P). The Leverage Plots represent plots of leverage of summed knee rOA progression (x axis) *vs* the baseline concentrations (y axis) of endo-EV and exo-EV IFN-γ **(C)** and IL-6 **(D)**. Endo-EV, within EV; Exo-EV, outside EV; HC, healthy control; OA-P, radiographic knee osteoarthritis progressor; OA-NP, radiographic knee osteoarthritis non-progressor.

Endo-EV IFN-γ concentrations were significantly higher in OA-P than both OA-NP and HC participants; exo-EV IFN-γ concentrations did not differ among the study groups (**Figure 4A**). In contrast, exo-EV IL-6 concentrations were significantly higher in OA-P than both OA-NP and HC participants; endo-EV IL-6 concentrations did not differ among the study groups (**Figure 4B**). There were no statistically significant differences between the study groups based on concentrations of endo-EV or exo-EV IL-1β, or iMFIs of IL-1β, IL-6 or IFN-γ in EVs of all sizes (data not shown). Adjusting for baseline clinical variables, baseline concentrations of endo-EV IFN-γ (**Figure 4C**) and exo-EV IL-6 (**Figure 4D**) were statistically significantly associated with knee rOA progression and yielded validated AUCs of 0.558, and 0.518, respectively.

## Discussion

All major types of immune cells (neutrophils, macrophages, NK cells, T cells, B cells, dendritic cells, and granulocytes) actively infiltrate OA synovial tissues, releasing cytokines and EVs into the joint that may contribute to structural progression of OA (11, 42–45). In this study, we identified plasma EVs from OA participants carrying surface markers indicative of multiple cell origins including human stem cells and progenitor cells, all major types of immune cells, activated pro-inflammatory fibroblasts, epithelial and endothelial cells (4, 15, 17–25). We identified plasma EVs in OA that carry the major pro-inflammatory cytokines, TNF-α, IFN-γ, IL-6 and IL-1β, demonstrating their pro-inflammatory phenotype. We found that the concentration of endo-EV TNF-α and the iMFI of TNF-α in EV subsets in OA plasma were associated with and strong predictors of knee rOA progression, while plasma exo-EV TNF-α was neither associated with nor predictive of knee rOA progression. Based on this prior work, we know that these TNF-α^+^ EVs are present and abundant in OA synovial fluid (15). This suggests that EV associated TNF-α may play a role in OA progression.

TNF-α is a classical pro-inflammatory cytokine produced by a broad variety of cell types, including, but not limited to, macrophages, monocytes, T cells, B cells, NK cells, mast cells, keratinocytes, astrocytes, microglial cells, muscle cells, intestinal paneth cells, tumor cells, synoviocytes and articular chondrocytes (46–48). Based on human *in vitro* data, TNF-α may play a critical role in the pathogenesis of OA (38, 40, 49). Previous human studies reported that serum TNF-α concentrations predicted knee rOA progression (50), and were associated with joint space narrowing (51). SF TNF-α concentrations were associated with knee pain (29). In addition, TNF-α polymorphisms were associated with susceptibility to OA in a Korean population (52).

Although in an animal study, TNF-α inhibition significantly decreased pro-inflammatory cytokines (IL-1β, IL-17a and IL-8), MMP-3 and MMP-9, inflammatory cell infiltration and bone destruction in joints and cartilage of rats with OA (53), the reported human trials of TNF-α inhibitors for OA are limited to three and all have been negative. These three trials (n=60 hand OA, n=84 hand OA, and n=20 knee OA) all used human TNF antibody, adalimumab (54). Only a single case report indicated that neutralizing TNF-α using adalimumab (40 mg subcutaneously every other week) decreased pain and improved joint function, visibly decreased synovial effusion and synovitis and bone marrow edema, and dramatically decreased nocturnal pain and improved walking distance (48). One possible explanation for these conflicting results is that TNF-α binds to two types of receptors with opposing functions — TNFRI mediating pro-inflammatory signals, and TNFRII mediating anti-inflammatory signals. Therefore, neutralization of TNF-α could result in a mixed clinical response, similar to prior observations resulting from treatments targeting both receptors: inhibiting signaling of both TNFRI and TNFRII reduced collagen-induced arthritis, but increased pro-inflammatory cytokine levels and reduced Treg cell activity. In contrast, selective blockade of TNFRI signaling reduced collagen-induced arthritis without the major side effects observed with both TNFRI and TNFRII blockade (55, 56). Another possible explanation is that TNF-α carried by EVs is sequestered from systemic anti-TNF-α. For flow cytometric quantification of intra-vesicular cytokines including TNF-α, it is necessary to fix and permeabilize the EVs; thus, permeability to antibodies is not expected to be a property of circulating EVs *in vivo*. We observed that the concentration of endo-EV TNF-α was higher than the matched exo-EV TNF-α in the same unit volume of OA plasma. Therefore, neutralizing soluble TNF-α in blood may not be sufficient when a large amount of pathogenic TNF-α is sequestered in EVs and potentially inaccessible to the neutralizing antibody. Nevertheless, these EVs would be expected to be able to fuse their membrane to the plasma membrane of specific target cells, followed by discharge of their luminal cargo [e.g. TNF-α) to the cytoplasm of their target cell (57)]. Given the dual biological functions of TNF-α in arthritis mediated by TNFRI and TNFRII, and potential technical difficulties depleting EV-carried TNF-α *in vivo*, neutralizing TNF-α with biologics may not be an ideal therapeutic approach for treating OA. Instead, it may be important to lower the production of TNF-α^+^ EVs. Monitoring of TNF-α^+^ EVs in plasma could also be a promising companion diagnostic for OA.

Similar to TNF-α, the concentration of endo-EV, but not exo-EV, IFN-γ was significantly associated with knee OA progression, although it was not as strong a predictor of knee OA progression. In contrast, the concentration of exo-EV, but not endo-EV, IL-6 was significantly associated with knee OA progression. These findings suggest that endo-EV and exo-EV pro-inflammatory cytokines may play different roles in the pathogenesis and worsening of OA and represent different biological processes in OA progression.

Among the test surface markers, CD56^+^ MEVs, related to NK cells, were the strongest predictor of knee rOA progression with the highest AUC. NK cells are one of the principal leukocyte subsets that infiltrate OA synovia (58); NK cells in both peripherial blood and SF of patients with OA produce pro-inflammatory cytokines,TNF-α, IL-6, and IFN-γ. The frequency of NK cells producing these pro-inflammatory cytokines is significantly higher in OA SF than plasma (59). *In vivo* antibody-mediated depletion of NK cells ameliorates disease in experimental OA, demonstrating their pathogenicity, which is likely mediated by their ability to promote inflammation and bone destruction (60).

The neutrophil to lymphocyte ratio (NLR) in peripheral blood is already a non-invasive and cost-effective biomarker of various systemic diseases, including cancers, cardiovascular diseases and rheumatologic diseases, and has been shown to be associated with OA severity (61–63). Recently, we found that the ratios of neutrophil EVs to lymphocyte EVs (from T cells, B cells and NK cells), and to T cell EVs, were highly correlated between SF and plasma in OA (15). Here we show that the ratio of neutrophil EVs to T cell EVs was associated with knee OA progression (with a validated AUC). These data taken together suggest that these NLR ratios, based on EV quantification, could serve as systemic biomarkers of OA joint pathology and possibly other comorbidities, such as cardiovascular disease. The breakthrough advantage of determining NLR ratio based on EVs is the ability to use frozen archival samples in contrast to the current method requiring fresh cells.

The first limitation of this exploratory study was the limited number of available samples related to difficulties collecting human specimens of matched gender, race, decade of age, and diversity of OA disease severity and progression. The second limitation was the lack of fresh cells, so we could not directly link EV profiles to the inflammatory phenotype of their parent cells. In addition, OA is often associated with comorbidities that might increase the prevalence of pro-inflammatory EVs. Given the sample sizes, assessing effects of comorbidities was outside the scope of these analyses. However, the GOGO study recruited cognitively intact, ambulatory older adults. In this regard, there is potential selection bias for individuals without major comorbidities other than OA. Although these limitations exist, this study identified several potential new knee OA progression-associated EV biomarkers and predictors worthy of further study as new systemic predictors of knee OA progression. This study also provides insights to optimize therapies targeting TNF-α pathways in OA. Our findings would encourage more studies to explore the roles of these EV biomarkers in OA pathogenesis and disease development.

In summary, we identified several immune cell- and pro-inflammatory cytokine-associated EV subpopulations that were significant independent predictors of knee rOA progression. Among these EV biomarkers, baseline iMFI of TNF-α in MEVs and SEVs was significantly higher in plasma of OA-Progressors than OA-Non-progressors, and the best predictor of knee rOA progression with highest AUC and specificity. Interestingly, baseline iMFI of TNF-α in EVs of all sizes, and total concentration of endo-EV TNF-α all predicted rOA progression. In contrast, baseline exo-EV TNF-α concentration was not associated with nor predictive of rOA progression. These data suggest that EV-associated TNF-α may be pathogenic in OA. Sequestration of large amounts of TNF-α in plasma EVs of OA patients, as shown here, may explain the disappointing results to date of TNF-α inhibitors in OA disease modifying trials.

## Data Availability Statement

The original contributions presented in the study are included in the article/**Supplementary Material**. Further inquiries can be directed to the corresponding author.

## Ethics Statement

The plasma samples used for this study were acquired under approval of Duke University (n=46) or acquired through a commercial entity, ZenBio (n=6) who provided an assurance, as indicated on their website (https://www.zen-bio.com/products/cells/), that all samples are acquired under donor consent and IRB approval. The patients/participants provided their written informed consent to participate in this study.

## Author Contributions

XZ and VK designed the study. XZ and JH performed the experiments and analyzed the data. M-FH performed ROC analyses. VK performed data analyses for radiographic procedures and grading. XZ and VK drafted the manuscript. All authors contributed to the article and approved the submitted version.

## Funding

This research was funded by the National Institute on Aging at National Institutes of Health, grant numbers R56AG060895 (VK and XZ), R01AG070146 (VK, XZ, and JH) and P30AG028716 (VK and JH).

## Conflict of Interest

The authors declare that the research was conducted in the absence of any commercial or financial relationships that could be construed as a potential conflict of interest.

## Publisher’s Note

All claims expressed in this article are solely those of the authors and do not necessarily represent those of their affiliated organizations, or those of the publisher, the editors and the reviewers. Any product that may be evaluated in this article, or claim that may be made by its manufacturer, is not guaranteed or endorsed by the publisher.
