# Supplementary material for: TNF-α Carried by Plasma Extracellular Vesicles Predicts Knee Osteoarthritis Progression
Source: Front Immunol. 2021 Oct 6;12:758386. doi: 10.3389/fimmu.2021.758386 (PMC8526961; doi:10.3389/fimmu.2021.758386)
Supplement: Supplementary file 1 [file DataSheet_1.docx]

**Supplementary Figure 1**

**
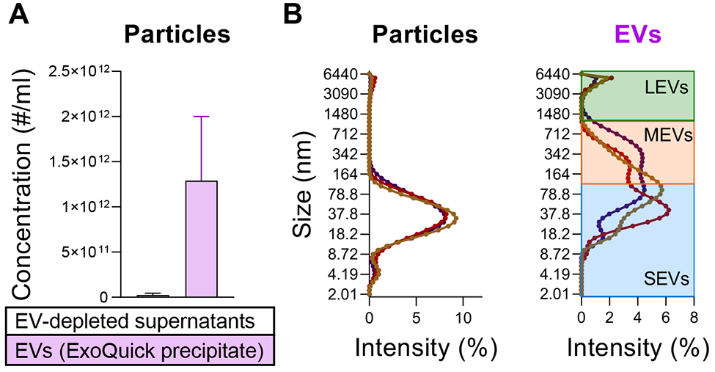
**

**Supplementary Figure 1** Nanoparticle tracking analysis (NTA) and dynamic light scattering (DLS) confirmed efficient EV precipitation by ExoQuick. The EVs and EV-depleted supernatants were separated from plasma of healthy controls (n=3). (A) The concentration of EVs and detectable particles in EV-depleted supernatants of plasma were measured by NTA. NTA data demonstrated that the number of detectable particles in the EV-depleted supernatants was between 0.08% - 5.84% of the number of particles (EVs) in the corresponding ExoQuick precipitates. (B) The size distribution of EVs and detectable particles in EV-depleted supernatants of plasma were measured by DLS. DLS data demonstrated that the size distribution of plasma EVs was from 2 nm to 6500 nm, while the size of the majority of detectable particles in EV-depleted supernatants of plasma was below 100 nm.

**Supplementary Figure 2**

**
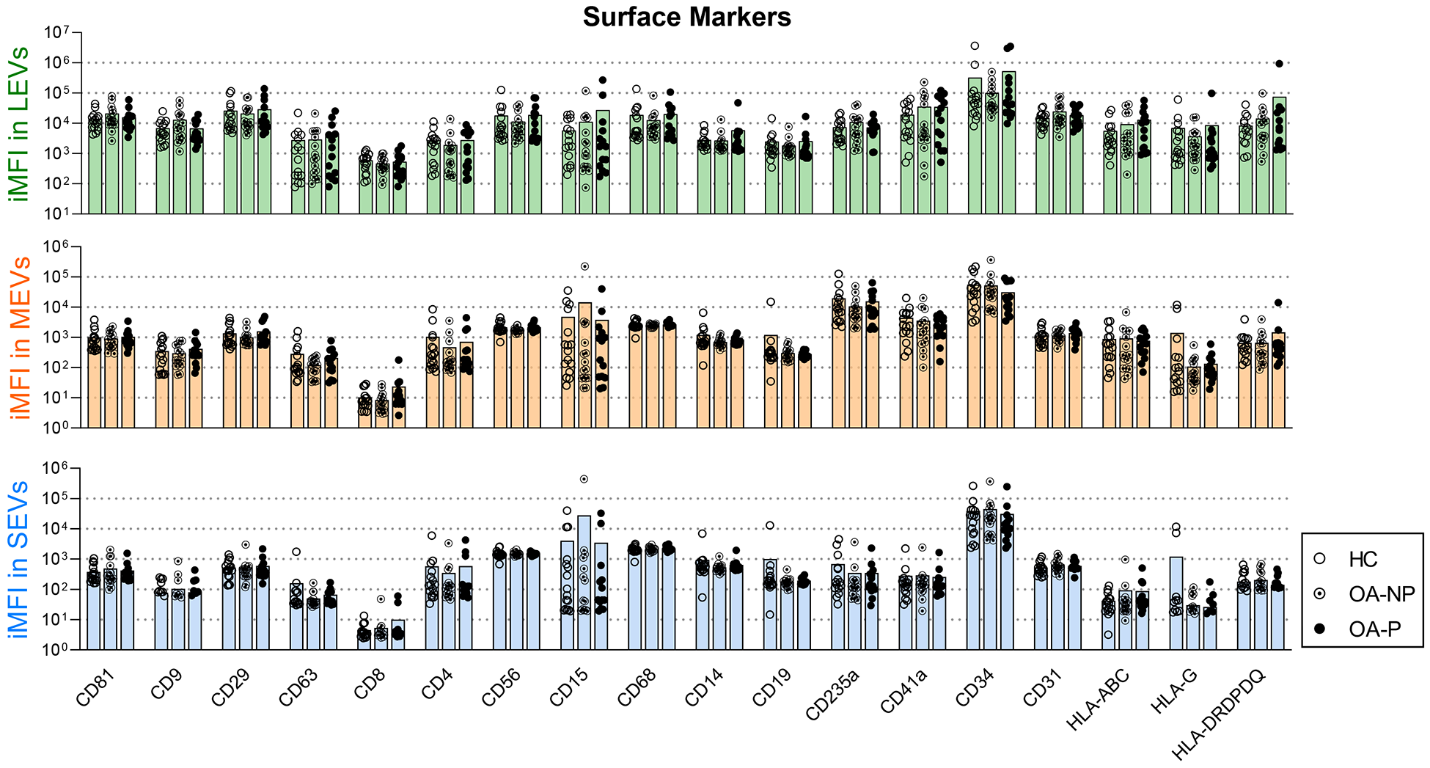
**

**Supplementary Figure 2** Plasma EVs from HC and OA participants all carried surface markers indicative of their cell origins from human stem cells and progenitor cells, immune cells, epithelial and endothelial cells. Plasma EVs from HC (n=16), OA-NP (n=16) and OA-P (n=14) participants at baseline were profiled with the indicated surface markers by high resolution multicolor flow cytometry. The graphs present a summary of iMFI of each surface marker in gated LEVs, MEVs or SEVs. Each dot represents a separate individual. Undetectable markers with “0” values were not plotted in the log scale, but included in the analysis. Comparisons between HC, OA-NP and OA-P were performed using Kruskal-Wallis test with significant results defined by FDR q< 0.05; none of the tested markers reached statistical significance. LEVs: large EVs; MEVs: medium EVs; SEVs: small EVs; iMFI: integrated mean fluorescence intensity. HC, healthy control; OA-P, radiographic knee osteoarthritis progressor; OA-NP, radiographic knee osteoarthritis non-progressor.

**Supplementary Figure 3**

**
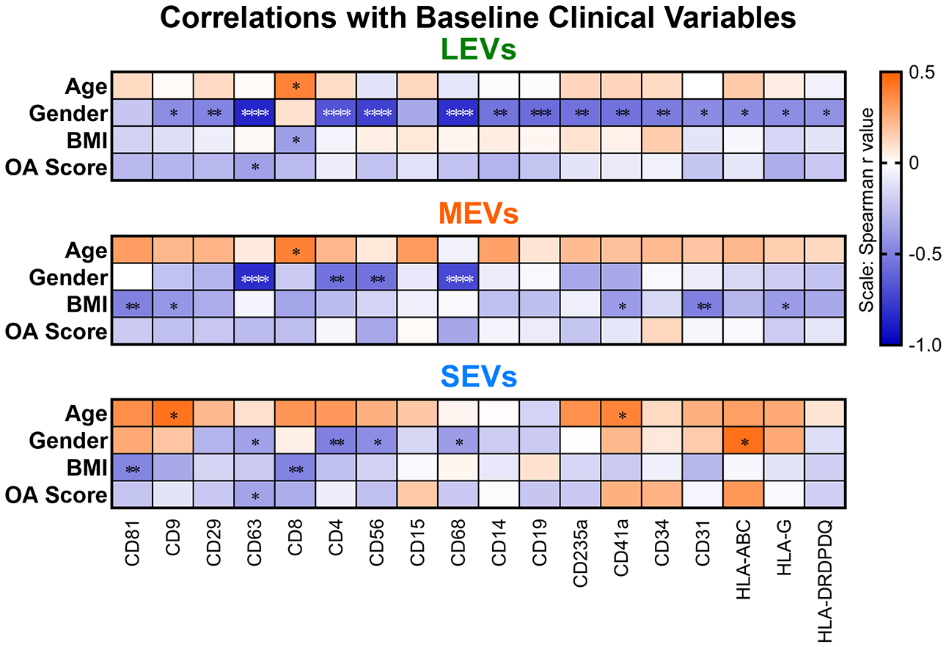
**

**Supplementary Figure 3** Subpopulations of EVs correlated with baseline clinical variables. EVs from plasma of OA participants (n=30) were profiled with the indicated surface markers in gated LEVs, MEVs or SEVs by high-resolution multicolor flow cytometry. Spearman correlation was used for assessing correlations between the baseline clinical variables (age, gender, BMI and summed knee OA K/L score) and baseline iMFI of individual surface markers in gated LEVs, MEVs and SEVs. The iMFI of several plasma EVs was positively correlated with baseline clinical variables, age, gender, BMI and summed knee OA radiographic severity score. Gender was defined as “0” for female and “1” as male. The heat maps were generated using the Spearman correlation coefficient r value; asterisks indicate the p value as follows: * <0.05, ** <0.01, *** <0.001, and **** <0.0001. LEVs: large EVs; MEVs: medium EVs; SEVs: small EVs.

**Supplementary Figure 4**

**
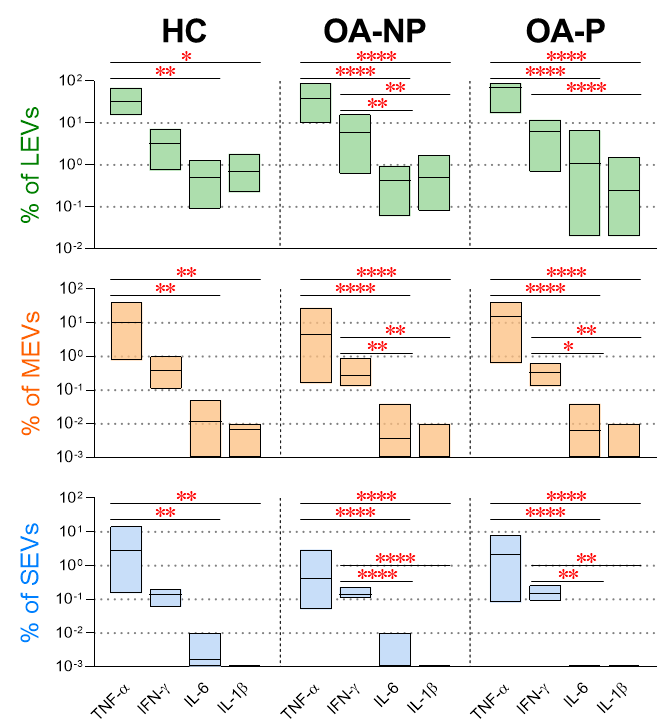
**

**Supplementary Figure 4** TNF-α was present in all sizes of plasma EVs and was the most abundant cytokine in EVs of all sizes and in all three participant groups. Plasma EVs from HC (n=6), OA-NP (n=16) and OA-P (n=14) participants at baseline were profiled for intra-vesicle TNF-α by high-resolution multicolor flow cytometry. The graphs present a summary of the percentage of EVs carrying the indicated cytokines in gated LEVs, MEVs or SEVs. Comparisons between the tested cytokines in each participant group were performed using Friedman test with significant results defined by FDR q< 0.05; asterisks indicate the p value as * <0.05, **p <0.01, ****p <0.0001. LEVs: large EVs; MEVs: medium EVs; SEVs: small EVs; HC, healthy control obtained from Zenbio; OA-P, radiographic knee osteoarthritis progressor; OA-NP, radiographic knee osteoarthritis non-progressor.
