# Supplementary material for: TNF-α Carried by Plasma Extracellular Vesicles Predicts Knee Osteoarthritis Progression
Source: Front Immunol. 2021 Oct 6;12:758386. doi: 10.3389/fimmu.2021.758386 (PMC8526961; doi:10.3389/fimmu.2021.758386)
Supplement: Supplementary file 2 [file Table_1.docx]

**Supplementary Table 1** Tested surface markers and major cell populations known to express them.

| Surface Markers | Major Cell Origin |
| --- | --- |
| CD9 | HSCs, B cells, T cells, NK cells, APCs, epithelial cells, endothelial cells |
| CD81 | HSCs, B cells, T cells, NK cells, APCs, epithelial cells, endothelial cells |
| CD29 | ASCs, MSCs, T cells, B cells, NK cells, APCs, neutrophils, endothelial cells, epithelial cells |
| CD63 | T cells, NK cells, platelets, basophils |
| CD8 | Cytotoxic T cells |
| CD4 | Helper T cells |
| CD56 | NK cells |
| CD15 | Neutrophils |
| CD68 | Macrophages |
| CD14 | Monocytes, macrophages |
| CD19 | B cells |
| CD235a | Red blood cells |
| CD41a | Megakaryocyte, platelets, HSCs |
| CD31 | HSCs, T cells, B cells, NK cells, APCs, endothelial cells |
| CD34 | HSCs, progenitor cells, endothelial cells |
| HLA-ABC | Nucleated cells and platelets |
| HLA-G | Monocytes, MSCs |
| HLA-DRDPDQ | APCs, activated T cells and pro-inflammatory fibroblasts |

HSCs: hematopoietic stem cells; ASCs: adipose stem cells; MSCs, mesenchymal stem cells; NK cells: natural killer cells; APCs: antigen presenting cells (including monocytes, macrophages and dendritic cells); HLA-ABC: HLA-A, HLA-B and HLA-C; HLA-DRDPDQ: HLA-DR, -DP and -DQ.
